# The Diagnostic and Therapeutic Value of Multimarker Analysis in Heart Failure. An Approach to Biomarker-Targeted Therapy

**DOI:** 10.3389/fcvm.2020.579567

**Published:** 2020-12-04

**Authors:** Albert Topf, Moritz Mirna, Bernhard Ohnewein, Peter Jirak, Kristen Kopp, Dzeneta Fejzic, Michael Haslinger, Lukas J. Motloch, Uta C. Hoppe, Alexander Berezin, Michael Lichtenauer

**Affiliations:** ^1^Department of Cardiology, Clinic of Internal Medicine II, Paracelsus Medical University of Salzburg, Salzburg, Austria; ^2^Internal Medicine Department, State Medical University, Zaporozhye, Ukraine

**Keywords:** biomarkers, heart failure, cardiovascular medicine, targeted therapy, multimarker analysis

## Abstract

**Background:** Heart failure is a pathophysiological state, which is still associated with high morbidity and mortality despite established therapies. Diverse well-known biomarkers fail to assess the variety of individual pathophysiology in the context of heart failure.

**Methods:** An analysis of prospective, multimarker-specific therapeutic approaches to heart failure based on studies in current literature was performed. A total of 159 screened publications in the field of biomarkers in heart failure were hand-selected and found to be eligible for this study by a team of experts.

**Results:** Established biomarkers of the inflammatory axis, matrix remodeling, fibrosis and oxidative stress axis, as well as potential therapeutic interventions were investigated. Interaction with end organs, such as cardio-hepatic, cardio-renal and cardio-gastrointestinal interactions show the complexity of the syndrome and could be of further therapeutic value. MicroRNAs are involved in a wide variety of physiologic and pathophysiologic processes in heart failure and could be useful in diagnostic as well as therapeutic setting.

**Conclusion:** Based on our analysis by a biomarker-driven approach in heart failure therapy, patients could be treated more specifically in long term with a consideration of different aspects of heart failure. New studies evaluating a multimarker – based therapeutic approach could lead in a decrease in the morbidity and mortality of heart failure patients.

## Introduction

Heart failure is a complex syndrome with major impact on public health. In the industrial world, about one out of ten persons over 65 years old is affected. Despite cardio-protective treatment with renin-angiotensin-aldosterone (RAAS) inhibitors, betablockers, neprilysin inhibitors and cardiac resynchronization therapy, mortality and hospitalization rates remain high (1). The wide introduction of implantable cardioverter defibrillators has minimized the risk of sudden cardiac death (2). Nevertheless, morbidity is persistent at a high level, with one out of four patients being rehospitalized within 30 days and almost half within 1 year of diagnosis (3, 4).

The best-studied biomarker in heart failure is B-type natriuretic peptide (BNP), which is released in response to increased left ventricular (LV) filling pressures and wall stress. BNP is broadly used in the diagnosis of clinical heart failure (5). Furthermore, BNP is used to guide the therapy of heart failure and left ventricular dysfunction in daily routine (6).

**Figure 1:**
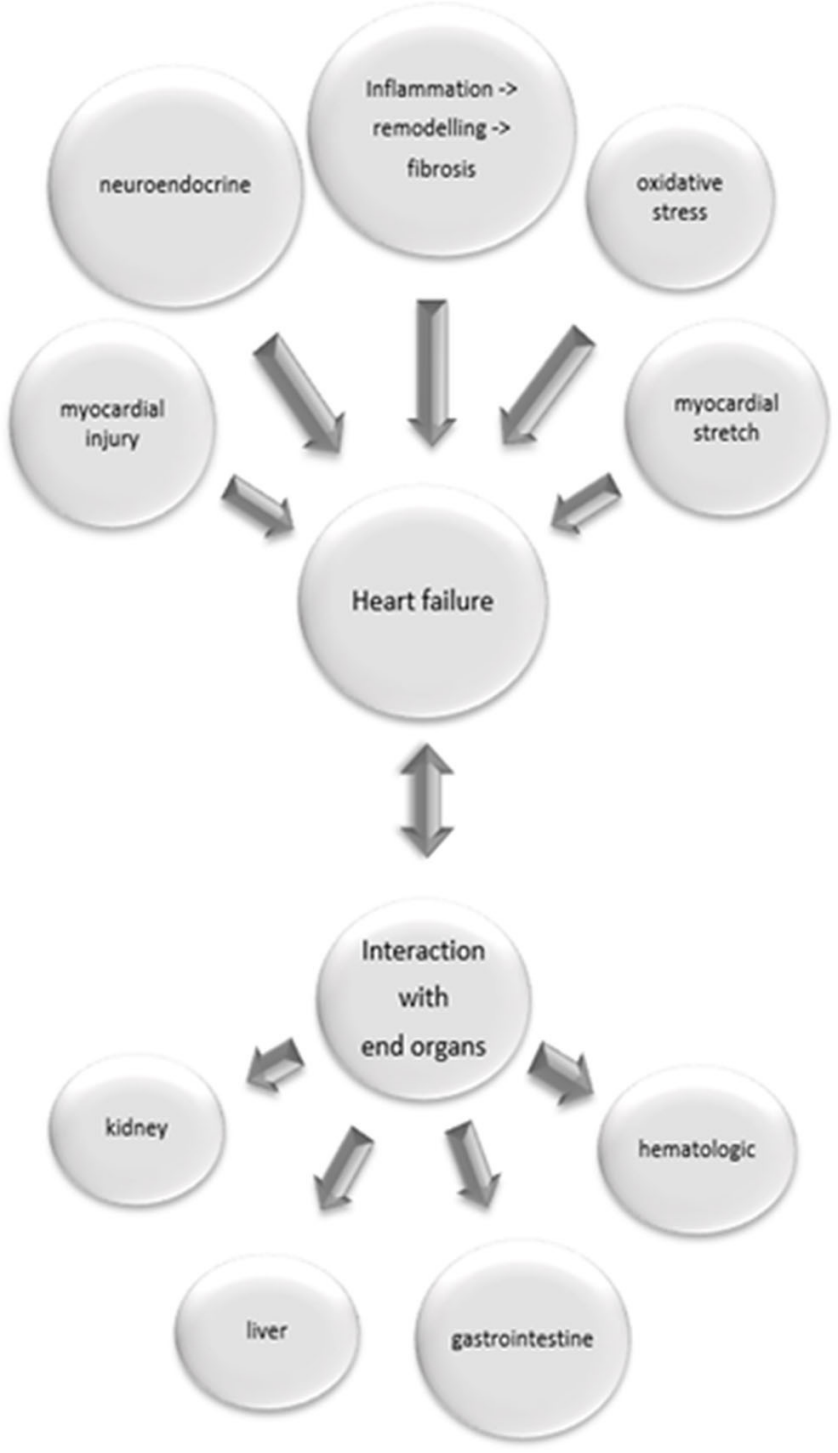
Pathophysiological processes relevant in heart failure and their interaction with end organs. Inflammatory processes with following remodeling and fibrosis, neuroendocrine activation, myocardial injury, oxidative stress and myocardial stretch contribute to heart failure. Heart failure might cause damage in end organs, but on the other hand failure of end organs induces heart failure.

The established biomarkers in heart failure fail to assess the variety of pathophysiological processes relevant to the condition of a heart failure patient (see Figure 1). Multi-marker analysis provides both a more valid prognostic stratification, and better evaluation of the patient's response to a therapeutic intervention (7). Due to emergence of more detailed information regarding the complex pathophysiological background of heart failure, a variety of biomarkers and new therapeutic interventions are currently under investigation.

In the following review, we present a prospective multi-marker-based therapeutic approach (Table 1) to the management of heart failure with a potential to decrease the roughly unchanged mortality and morbidity of heart failure.

**Table 1:** Cardiac biomarkers, described in the report, and their diagnostic function in the pathophysiology of heart failure.

**Influence on heart failure**	**Biomarker**
Myocardial injury	Troponin, H-FABP
Inflammation	CRP, IL-6, TNF-α, IL-1-Beta,
Remodeling	sST-2, Galectin,
Fibrosis	TGF-ß
Mechanical stretch	BNP, GDF-15
Neurohumoral	Copeptin, Endothelin-1,
Oxidative stress	Uric acid, Myeloperoxidase,
Micro RNA	miR-18a-5p, miR-26b-5p, miR-27a-3p, miR-30e-5p, miR-106a-5p, miR-199a-3p, miR-652-3p, miR-30c, miR-221, miR-328, miR-375, miR-423, miR-34a, miR-21-3p,miR-199, miR-30a

**Figure 2:**
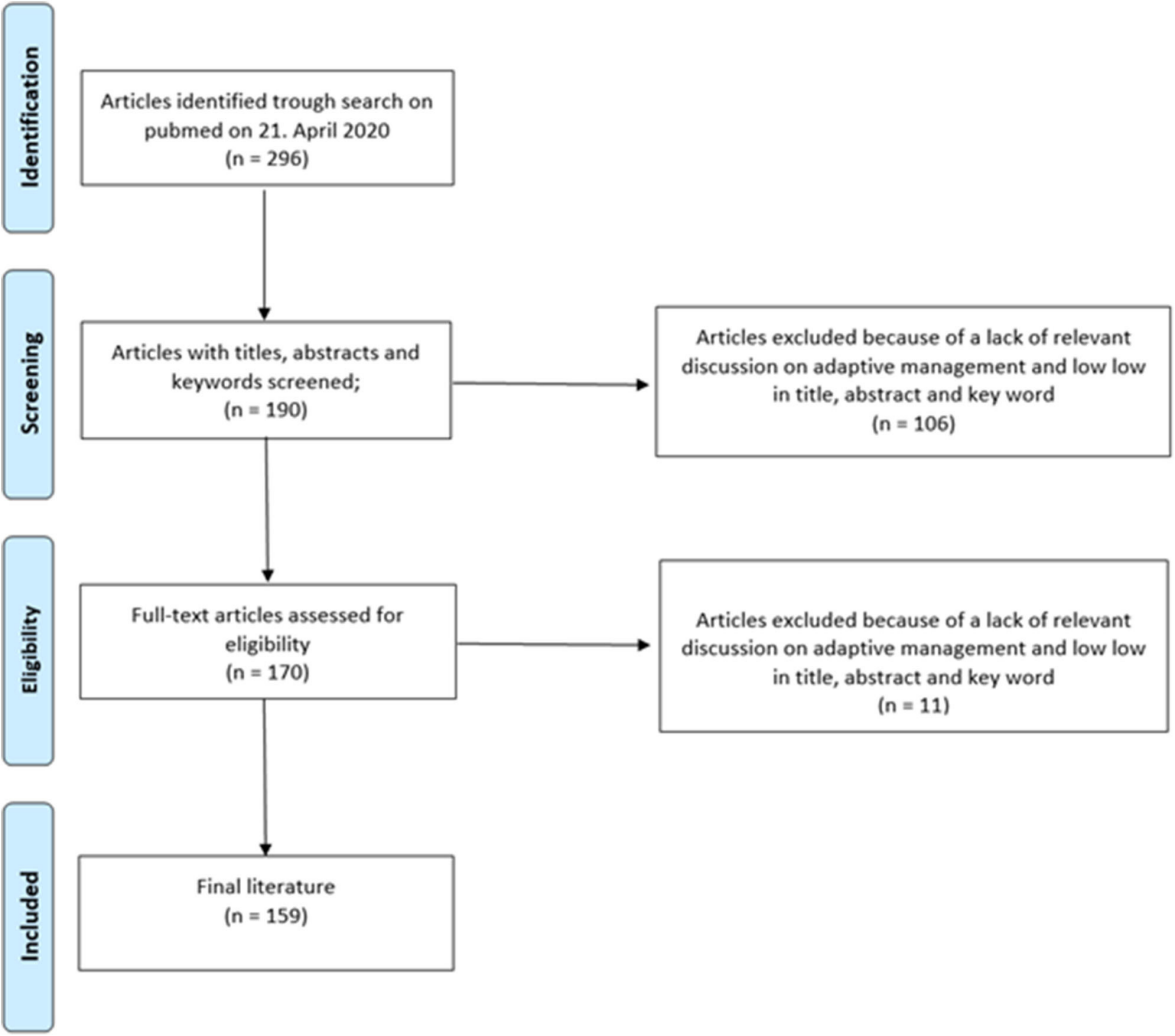
Flowchart outlining the protocol adopted in this systematic review for literature selection.

An analysis of prospective, multimarker-specific therapeutic approaches to heart failure based on studies in current literature was performed. A total of 296 screened publications in the field of biomarkers in heart failure were screened and 159 were found to be eligible for this study due to the relevance of their contribution to this topic by a team of experts (Figure 2).

## Biomarkers of the Inflammatory/Matrix Remodeling/Fibrosis Axis

### Inflammation

The role of inflammation in heart failure has been proposed as levels of inflammatory cytokines including tumor necrosis factor (TNF), interleukin (IL)-1β and IL-6 were found to be elevated in heart failure. TNF and IL-6 serum levels correlate with heart failure severity and prognosis. Elevated basal cytokine levels might even predict future development of heart failure (8).

Renal failure, arterial hypertension, chronic obstructive pulmonary disease (COPD), diabetes mellitus, metabolic syndrome and myocyte injury following myocardial infarction or viral infection are suspected to result in chronic inflammatory processes, which may contribute to the ten percent increase in heart failure incidence in the industrialized world (9). Consequently, attention has focused on the role of inflammation in heart failure, in particular due to an increasing number of immunosuppressive therapeutic opportunities. Biomarkers, identifying inflammatory processes in heart failure, are becoming recognized as indicator of inappropriate immune response and for potential therapeutic value (10).

CRP represents a composite heart failure biomarker, which is a robust predictor of cardiovascular and non-cardiovascular mortality. Interestingly, an increased hsCRP was associated with a higher cardiovascular (CV) mortality irrespective of the baseline ejection fraction (11). Non-immunosuppressive therapies, including β-blockade, renin-angiotensin antagonists and statins had modest effect on reducing CRP levels (12). CRP, as an acute phase protein synthesized by hepatocytes, is induced by IL-6 signaling which itself is activated by an up-regulation of IL-1 β and TNF (13). This signal cascade offers further therapeutic possibilities.

Consequently, IL-1 β, as a biomarker and respective signal point with a potential of targeted heart failure therapy, is gaining attention. In animal models, a direct influence of IL-1β injection on systolic function was shown as well as on diastolic function (14). In acute heart failure, IL-1β could identify patients with high 1-year mortality (15). These results are hypothesis generating. Whether biomarker-guided therapy is beneficial requires further studies, especially after well-established biomarkers, including NT-pro-BNP, have failed to improve therapy results. However, an 84% reduction in CRP levels was seen in an initial open label-study with Anakinra therapy in seven patients with acute heart failure (16). These promising results led to the first randomized controlled trial (RCT) with Anakinra in 30 patients with acute heart failure, in which Anakinra again proved to reduce inflammatory response (17). In a separate study of 60 patients with acute heart failure, Anakinra was started 2 weeks after discharge and continued for 12 weeks, and improved peak oxygen consumption, quality- of-life, and NT-pro-BNP levels (18). These results were partly reproduced in an RCT in 30 patients with heart failure with preserved ejection fraction (HFpEF), in which significant improvement in quality-of-life, treadmill exercise time, and a reduction in NT-pro-BNP levels were seen, yet here no significant changes in oxygen consumption were observed (19). The negative results in oxygen consumption, however, may perhaps be attributed to low cardiorespiratory fitness in patients with HFpEF, commonly associated with severe obesity. A recently published trial of 10,061 patients with heart failure under treatment with Canakinumab showed a significant reduction in the composite endpoint of hospitalization for heart failure and heart failure related mortality (20).

Heart failure leads to sympathetic activation, which is coupled with an elevated pro-inflammatory cytokine profile. Given the evidence that the pro-inflammatory cytokine TNFα and soluble TNF receptors correlate with mortality in heart failure, it is thought that targeting this mediator may be beneficial in stopping heart failure deterioration (21). The randomized-multicenter clinical trials RENAISSANCE and RECOVER used the genetically engineered humanized TNF receptor Etanercept to neutralize TNFα in patients with chronic heart failure (22). Surprisingly, patients with heart failure on Etanercept had a worse prognosis compared to placebo in the RENAISSANCE trial. Moreover, a worsening of heart failure was documented in the ATTACH study using infliximab therapy (23). Pentoxifylline, a promising immunomodulatory therapy, also inhibits TNFα and furthermore reduces programmed cell death in multiple cell types by downregulating the expression of the apoptosis-signaling surface receptor Fas/APO-1 (24, 25). A meta-analysis of six studies in patients with heart failure treated with pentoxifylline showed a 4-fold reduction in mortality, leading to optimism with respect to this therapy (26).

Another interesting strategy is the application of intravenous immunoglobulin (IVIG) which proves to influence cytokine production in T-cells and monocyte/macrophage both in *in-vitro* and *in-vivo* studies (27, 28). In a placebo-controlled clinical trial in forty patients with chronic heart failure using intravenous immunoglobulin (IVIG), a significant improvement of left ventricular ejection fraction and a reduction of N-terminal pro–atrial natriuretic peptide was demonstrated (29).

## Cardiac Remodeling

Cardiac remodeling is characterized by cellular and interstitial changes, usually in response to acute or chronic damage, consequently leading to changes in size, mass and function as well as geometry of the heart (30–33). In response to these mechanisms, the cardiac function decreases. Cardiac remodeling is usually induced by myocardial damage as for example in the context of myocardial infarction (34). However, also chronic inflammatory or subclinical ischemic processes as well as increased cardiac strain can lead to cellular and interstitial changes (9). Furthermore, remodeling is also promoted by an imbalance of metabolic pathways as for example in the leptin-neprilysin-aldosterone axis (35). Nevertheless, the stratification of the processes mentioned above remains challenging because the established cardiovascular biomarkers troponin and natriuretic peptides are not well suited to reflect the extent of cardiac remodeling. In this respect, novel cardiac biomarkers represent promising new modalities to further refine diagnosis and therapy monitoring.

Here, sST2, GDF-15 should be covered pars pro toto, as they represent some of the most established markers in the field. sST2 represents a marker predominantly used in the field of heart failure, where it was shown to independently predict mortality and hospitalization in acute or chronic heart failure (36, 37). There are two known isoforms of ST2, a soluble form (sST2) and a membrane bound form (ST2L). Through binding of IL-33, the only known ligand for ST2, cardio-protective effects can be mediated through the ST2L receptor (38). ST2L also enhances the functions of T-cells, mast cells and cells of the innate lymphoid type (39). On the other hand, sST2 acts as a decoy receptor for IL-33, thus preventing its potential beneficial effects resulting in myocardial hypertrophy and cardiac remodeling. Thus, sST2 was shown to be a strong predictor for mortality in acute heart failure at admission but also at discharge (40). Furthermore, with respect to chronic heart failure, a meta-analysis including over 6,000 patients was able to show that sST2 predicts all-cause mortality as well as cardiovascular mortality. A cut-off for sST2 below 35-ng/ml was associated with a significant improvement in chronic heart failure, regardless of clinical presentation (41). Furthermore, sST2 was also shown to be elevated in acute coronary syndrome, correlating with mortality rates after myocardial infarction (42–44). Given its pathophysiological background, elevated levels of sST2 were also reported in pulmonary hypertension as well as in peripheral artery disease (45, 46).

The cytokine GDF-15 is involved in the regulation of inflammation and fibrosis, and is expressed in most organ systems. Furthermore, GDF-15 is also involved in apoptotic processes. The secretion of GDF-15 is upregulated in response to organ damage. With respect to the cardiovascular system, GDF-15 induces cardio-protective effects through different cellular signaling pathways, interacting with ALK 1–7 and Smad-receptors, as well as the epidermal growth factor receptor (EGFR) and NF-κB/JNK/caspase-3 pathway (47). Elevated levels of GDF-15 have been reported in heart failure patients with an impact on mortality rates in heart failure with reduced and preserved ejection fraction (48). Furthermore, GDF-15 is also an independent marker of all-cause mortality and cardiovascular events in patients with coronary artery disease (49). Apart from cardiovascular disease entities, a predictive value of GDF-15 was also reported for other disease entities, with a focus on diabetes, malignancies and chronic kidney disease (50).

Thus, when considering different pathophysiological processes involved in cardiac remodeling, such as inflammation, increased cardiac strain and oxidative stress, the novel cardiac biomarkers sST2 and GDF-15 are a promising for a more sophisticated assessment of cardiac remodeling (51). Their reduced cardiac specificity and involvement in numerous organ systems, initially deemed a disadvantage, may turn out to be a strength with respect to their prognostic value.

## Cardiac Fibrosis

Most etiologies of heart disease cause a pathological myocardial remodeling resulting in a cardiac fibrosis (52). In contrast to other organs, the heart has restricted regenerative ability after damage. Physiologically, fibrotic processes serve to preserve the structure integrity and pressure-generating capacity of the heart. Otherwise a myocardial dysfunction or rupture might result (34, 53). Unlike pathological remodeling within the context of chronic cardiac inflammation processes, the excessive deposition of extracellular matrix results in deformed organ structure and an impairment of cardiac function (54). Fibrogenesis increases ventricular stiffness and can lead to contractile dysfunction. An excess of extracellular matrix worsens mechanical-electric coupling of cardiomyocytes with an impact on cardiac contraction and an increased risk of arrhythmogenesis and mortality (55, 56).

Transforming Growth Factor β 1 (TGF-β1) is perhaps the most extensively studied biomarker of fibroblast activation and is known to have an impact on fibrotic processes in many organs. TGF-β1 is initially released in a complex with latent TGF-β binding proteins that inhibit its activity. It can be activated by proteolytic cleavage, and by binding to an activin receptor-like kinase (ALK 5), it is able to activate pro-fibrotic genes (57). This process is called the canonical pathway (58). Inhibitors of the TGF-β receptor ALK5 are under analysis as presumed antifibrotic targets. ALK5 inhibitors can reduce TGF-β activity, rescuing cardiac dysfunction and facilitating the remodeling after a myocardial infarction (59). While inhibition of the canonical TGF-β signaling pathway seems promising, this concept needs further studies and refinements before it has an impact on clinical routine. TGF-β can also induce non-canonical signaling that involves several mitogen-activated protein kinases (MAPKs). This pathway activates TGF-β activated kinase (TAK) 1, which results in a pathological cardiac remodeling and consequently in heart failure (60). Supposedly, the non-canonical pathway may be the dominant process in cardiac fibrosis. Investigations to prevent this non-canonical signaling seem to be more promising for the treatment of cardiac fibrosis and heart failure. TAK1 might serve as a viable therapeutic target. Furthermore, the inhibition of p38 is being actively investigated for its antifibrotic potential (61).

**Figure 3:**
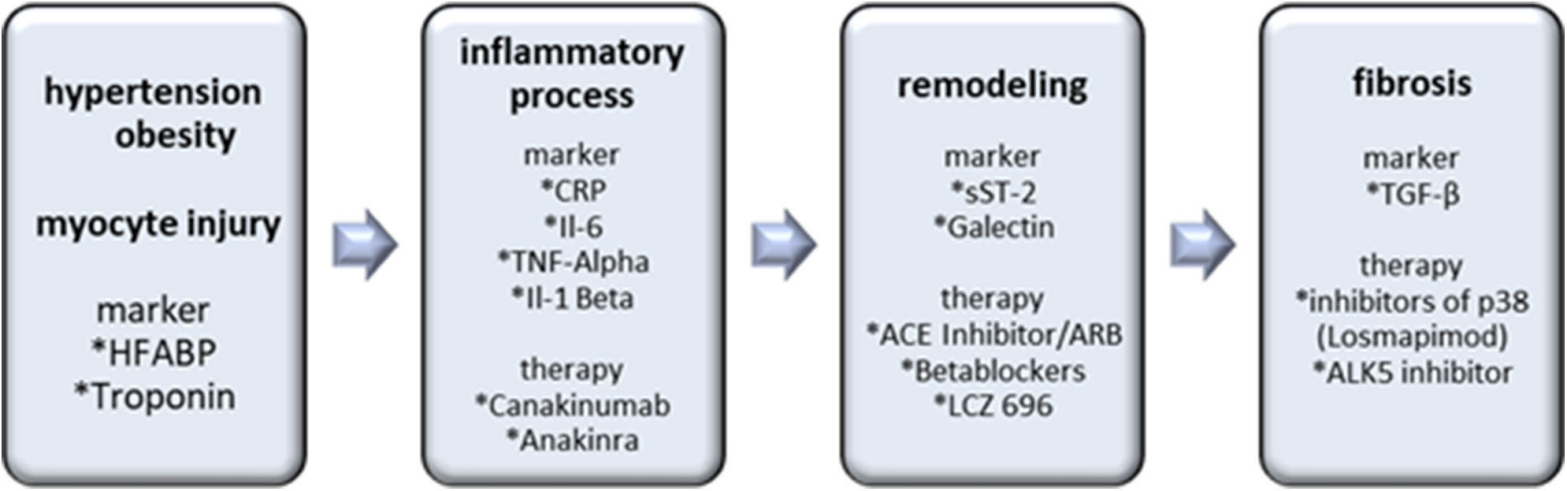
Biomarkers and therapy options in the inflammatory/matrix remodeling/fibrosis axis. Hypertension, obesity and myocyte injury cause inflammatory processes with following remodeling and fibrosis as an end result. Inflammatory markers are important for the detection and antibodies, including Canakinumab and Anakinra are prospective therapy options. sST-2 and Galectin indicate remodeling processes. The most important marker of cardiac fibrosis is TGF-ß. Losmapimod and ALK5 inhibitors are the most promising therapeutic interventions.

In the SOLSTICE Phase II trial, patients after a non – ST segment elevation myocardial infarction received the p38 inhibitor losmapimod without observation of major side effects. Although infarct size was non-significantly reduced, patients with intake of losmapimod experienced fewer cardiac events, such as heart failure. These findings provide hope for the therapeutic potential of losmapimod and resulted in the enrollment for the phase III LATITUDE-TIMI 60 trial. The primary endpoint, including a significant reduction of cardiovascular death, remained unmet, but in patients with a STEMI with higher age, elevated hs-CRP or chronic kidney disease, the risk of heart failure tended to be reduced (62, 63). Further studies evaluating the antifibrotic effects of p38 on negative cardiac outcomes are warranted (see Figure 3).

## Biomarkers of the Oxidative Stress Axis

**Oxidative stress** is involved in the development and progression of heart failure. Oxidative stress is defined as dysregulation between the production of reactive oxygen species (ROS) and the endogenous antioxidant defense mechanism system. ROS production is primarily caused by mitochondria, NADPH oxidases, xanthin oxidase and nitric oxide synthase, whereas the most important endogenous antioxidant defense mechanisms include superoxide dismutase (SOD), catalase, glutathione peroxidase (GPx), nicotinamide adenine dinucleotide (NAD+) and glutathione (64). Under pathological conditions, the electron transport chain of the mitochondria provokes the formation of large quantities of superoxide.

ROS production is enhanced in pathologic stimuli resulting from mechanical stretch, neurohumoral or inflammatory activation, and myocyte injury (65). Excess of ROS causes cellular dysfunction, protein and lipid peroxidation, DNA damage, and eventually leads to irreversible cell death. Furthermore, ROSC leads to maladaptive myocardial remodeling with subsequent myocardial fibrosis and to impairment of the electrophysiological and contractile apparatus by modification of crucial proteins in the process of excitation (66).

In acute myocardial infarction, leucocytes release myeloperoxidase (MPO), resulting in the generation of oxidizing species (67, 68). In epidemiological studies, a correlation of elevated MPO in chronic heart failure (CHF) was demonstrated even after adjustment for B-type natriuretic peptide (pBNP) or age (69). MPO in combination with CRP and pBNP showed a better rate of detection of left ventricular dysfunction than pBNP alone in a prospective study with 1,360 patients (70). However, there was no significant difference of MPO level in the setting of acute dyspnea in 667 patients with either acute heart failure or other non-cardiac dyspnea (71).

Furthermore, in a different prospective study evaluating in 412 patients with acute on chronic heart failure, the diagnostic accuracy of MPO could not be proven and showed no correlation with 1-year mortality (72).

Small trials stated the beneficial effect on improved myocardial function, peripheral vasodilatation capacity, reduced BNP and increased LV ejection fraction. In a larger trial enrolling 405 patients, oxypurinol in general failed to improve the clinical status, but in the subset of patients with elevated uric acid, oxypurinol improved the symptoms of heart failure. This study highlights the need for a careful selection of patients potentially benefitting from anti-oxidative therapy.

**Figure 4:**
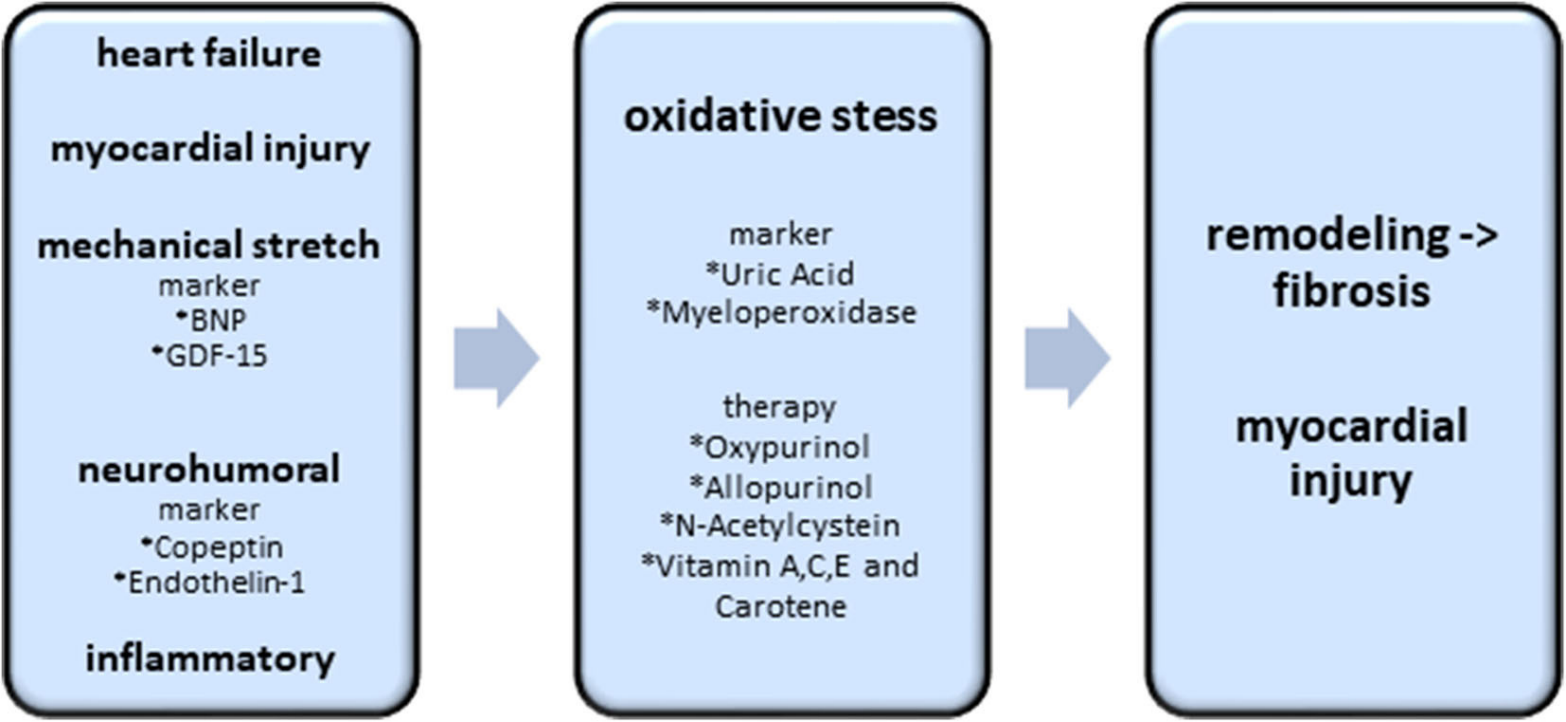
Biomarkers and potential therapy options of the oxidative stress axis. Existing heart failure, mechanical stretch, neurohumoral activation and inflammatory processes induce the excess of oxidative stress. Oxidative stress itself causes cardiac fibrosis and myocardial injury. The best investigated biomarkers, indicating an excess of oxidative stress, are uric acid and myeloperoxidase. Oxypurinol, Allopurinol, N-Acetylcystein and Vitamin A,C,E are the most promising therapeutic approaches.

**Figure 5:**
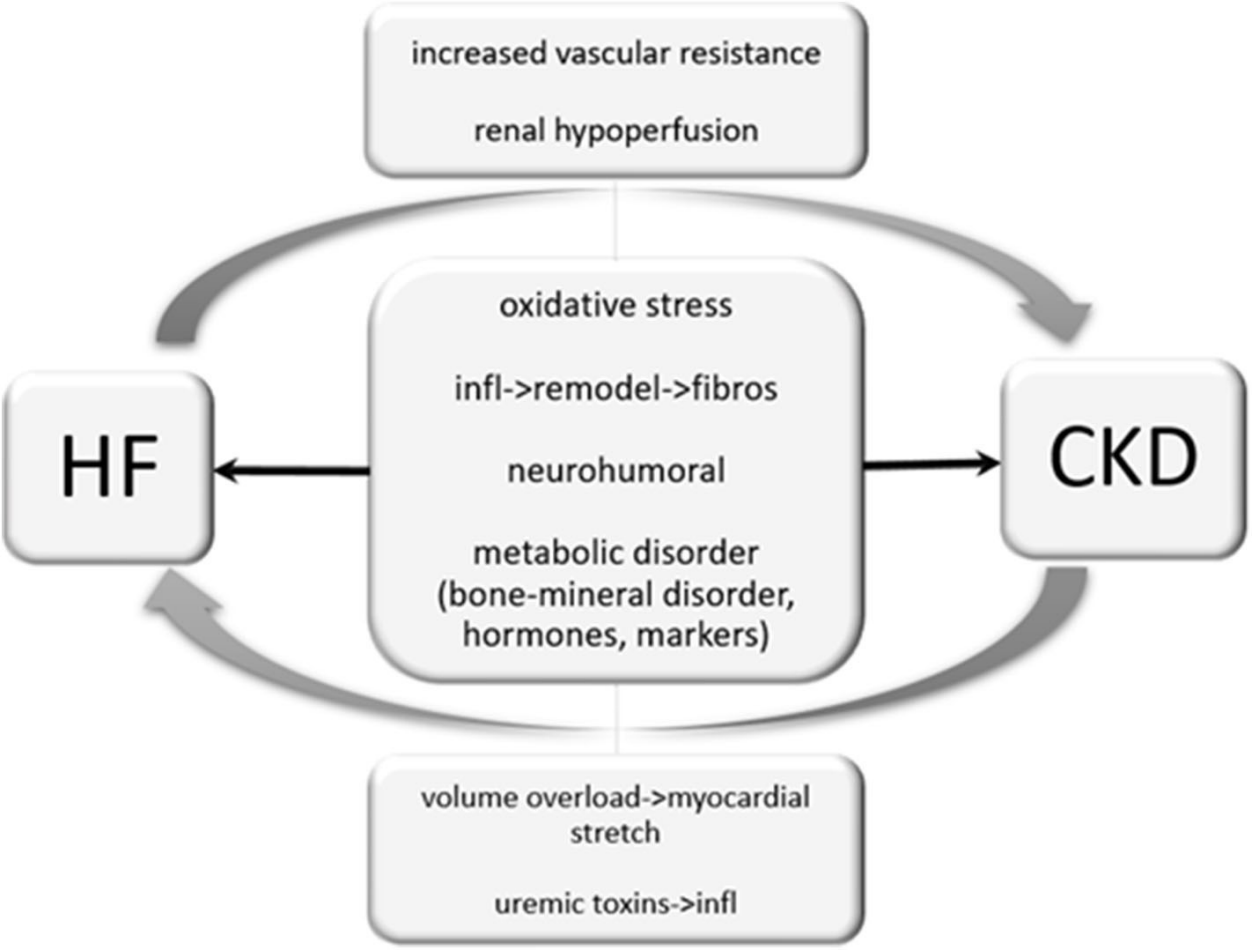
Cardio-renal interaction. Heart failure can cause renal hypoperfusion, increased vascular resistance and therefore results in an impairment of renal function. Unlike, chronic kidney disease may cause volume overload and results in a myocardial stretch. Uremic toxins provoke inflammatory processes and are reported to worsen heart failure. Oxidative stress, inflammatory processes and neurohumoral activation may independently by its origin impair cardiac and renal function.

The more extensively studied antioxidative stress therapy in patients with heart failure is the inhibition of xanthin oxidase using allopurinol or oxypurinol. Uric acid, produced by xanthin oxidase (XO) and representative for the activation of the enzyme XO has pro- and antioxidant effects (73, 74). Several studies investigated the correlation of uric acid and cardiovascular mortality. While in the majority of the studies, correlation between cardiovascular mortality or incidence of heart failure with uric acid level was significant, the Framingham Heart Study could not show any significant correlation (75). Interestingly, a systemic review and meta-analysis of eight studies evaluating the effect of xanthin oxidase inhibitors in cardiovascular diseases did not show a significant reduction in mortality compared to placebo (76). However, in a subgroup-analysis of patients with normal or mildly impaired kidney function, a significant reduction of all-cause mortality in heart failure was observed (77). This might reflect the problem of various mechanisms resulting in an elevation of uric acid (see Figures 4 and 5).

Taking this problem into account, selecting patients with elevated uric acid due to chronic inflammation rather than kidney failure might result in significant reduction of mortality. This thesis is supported by large trials showing a significant reduction of all-cause mortality in heart failure patients with a glomerular filtration rate ≥30 ml/min/1.73 m^2^ (78, 79). In trials investigating the antioxidative effect of vitamin A, C, E and folic acid, all failed to demonstrate a reduction in outcomes or progression of CV disease (80).

N-Acetylcysteine (NAC) is a thiol-containing antioxidant with the capacity to regenerate intracellular antioxidant pools. The NACIAM (NAC in Acute Myocardial Infarction) trial, which examines the use of high dose NAC in combination with low-dose nitroglycerin in ST-segment elevation myocardial infarction patients after percutaneous coronary intervention, also demonstrated that the combined use of NAC and nitroglycerin might significantly lower infarct size and increase myocardial salvage with a consequent less severe following heart failure (81).

Endogenous antioxidant therapy trials seem to have a promising future in the treatment of heart failure, but are still in an experimental phase (82).

## microRNAs in Heart Failure (HF)

Since their discovery in 1993 by Lee et al. (83), microRNAs (miRNAs) have gained increasing attention by the scientific community due to their diagnostic and therapeutic potential in various disease entities. MiRNAs are a class of small (19–24 nucleotides) ribonucleic acids (RNAs) with a paramount role in posttranslational gene silencing (PTGS). After enzymatic processing, the mature miRNA binds to RNA-induced silencing complexes (RISC), which then binds complementary messenger RNAs (mRNAs), thusly preventing protein synthesis (84, 85). MiRNAs can be found in all eukaryotic organisms and are probably involved in the regulation of a wide variety of physiologic and pathophysiologic processes (86). In fact, recent studies suggest that a variety of miRNAs play a role in pathophysiologic processes involved in the development and progression of heart failure (HF), such as cardiac remodeling, inflammation or myocardial fibrosis (87–89). Furthermore, miRNAs have been identified as useful additive diagnostic biomarkers in patients with cardiovascular diseases which could add discriminative value to established and commonly used biomarkers (90–92). Hence, the diagnostic and therapeutic potential of miRNAs in patients with HF is of scientific interest, and why it will be discussed briefly in the following.

### Diagnostics

Several studies have investigated expressed miRNA concentrations in patients with acute and chronic heart failure (93–95). For example, a recent study by Ovchinnikova et al. investigated the miRNA expression patterns of patients with acute heart failure (AHF) and chronic heart failure (CHF) and compared them with the patterns of patients with chronic obstructive pulmonary disease (COPD) and healthy controls. Interestingly, the authors could discriminate a panel of seven downregulated miRNAs in patients with AHF (miR-18a-5p, miR-26b-5p, miR-27a-3p, miR-30e-5p, miR-106a-5p, miR-199a-3p, and miR-652-3p), which were also associated with adverse outcomes, while there was no difference in miRNA expression between patients with COPD and healthy controls (96).

Besides, miRNAs could also be useful in differentiating heart failure with preserved ejection fraction (HFpEF) from heart failure with reduced ejection fraction (HFrEF). Hence, Watson et al. recently found that serum levels of miR-30c, miR-221, miR-328, and miR-375 could adequately discriminate HFpEF from HFrEF, especially when combined with the plasma concentrations of BNP (97).

In particular, miRNAs have a potential to distinguish heart failure of different etiologies and therefore, play an essential role on long term prognosis. Circulating levels of miR-423 and miR-34a were reported to be higher in patients with ischemic cardiomyopathy, whereas expression levels of miR-21-3p, miR-199 and miR-30a were rather elevated in the non-ischemic heart failure cohort. Furthermore, miR-423 is associated with a poorer prognosis in patients with acute HF (98).

Similarly, miR-29a was found to be significantly upregulated in patients with hypertrophic cardiomyopathy (99) and it could also discriminate hypertrophic obstructive cardiomyopathy (HOCM) from hypertrophic non-obstructive cardiomyopathy (HNCM) and aortic stenosis in a recent study (100). Based on these findings, miRNA-panels could be a useful additive diagnostic approach in the future and could facilitate diagnosis and risk stratification in patients with HF.

However, miRNAs have not only been investigated for their diagnostic utility in various disease entities, including cardiovascular diseases. In fact, several miRNAs have already been applied therapeutically in different diseases in humans, for example in patients with hepatitis C virus (HCV) or oncological diseases (101, 102). Regarding the known pathophysiologic processes underlying the development and progression of HF, miRNAs also may find therapeutic use in affected patients.

### Therapeutics

Inflammation and cardiac fibrosis are key features of cardiac remodeling, which is itself crucial for the pathogenesis and progression of HF. Recent evidence suggests, that inflammation and fibrosis are potentially reversible, rendering them interesting targets for novel therapeutic approaches in the management of HF. In fact, several miRNAs are known to interact with inflammatory, fibrotic and apoptotic pathways. For example, miR-21 was found to enhance myocardial fibrosis by targeting the extracellular signal-regulated kinase (ERK)–mitogen-activated protein (MAP) kinase pathway via sprouty homolog 1 (SPRY1) (103) and the phosphatase and tensin homolog (PTEN)–AKT phosphorylation-dependent pathway (104). Interestingly, silencing of miR-21 significantly ameliorated cardiac fibrosis and cardiac dysfunction in two recent animal models (105). Besides reducing myocardial fibrosis, silencing of miR-21 was also found to reduce the expression of the miRNA of RORγt, a crucial factor of T-cell development, thus decreasing myocardial inflammation in an animal model of CVB3-induced myocarditis (106). In contrast, miR-29b was recently found to be downregulated in mice with myocardial fibrosis, and overexpression led to an attenuation of fibrosis and cardiac dysfunction via the transforming growth factor (TGF)-β/Smad3 signaling pathway (107).

Based on the findings of recent animal models, various miRNAs could represent attractive targets for innovative therapeutic approaches in patients with HF, for example silencing of miR-21 or substitution of miR-29b. In fact, several phase 1 and 2 trials are currently being conducted in humans, investigating the application of miRNA-based therapies (108). The most advanced of these is miravirsen, a subcutaneously administered antagonist of miR-122, which is currently under investigation in patients with chronic hepatitis C and shows promising results with no relevant adverse effects. With the advent of miRNA-based therapies in humans, potential applications for the management of acute and chronic heart failure may arise. However, whether these approaches will impact our clinical practice in the future remains to be elucidated in large endpoint trials.

## Interaction with End Organs

### Cardio-Renal Interaction

Approximately 50% of patients with chronic heart failure have a chronic kidney disease (CKD) and 33–56% of HF patients have impaired renal function. CKD is associated with high mortality in patients with heart failure. Due to pathophysiological processes and interactions with each other, dysfunction of one organ may induce pathology in the other one (109). The most studied biomarker in cardio-renal syndrome is B-type natriuretic peptide, which only represents a single pathophysiological pathway in heart failure and is influenced by numerous factors, including renal function, aging, obesity, anemia, sepsis, hypertension, atrial fibrillation, diabetes mellitus, liver cirrhosis and cancer chemotherapy. Since the publication of the heart failure AHA guidelines 2013, the role of ST-2 and galectin in cardio-renal interaction in the context of heart failure has been evaluated. Especially in the presence of a CKD, ST-2 is of great interest. ST-2 is the biomarker least influenced by renal function. Besides the predictive role of Galectin-3 in heart failure in terms of morbidity and mortality, it may be causally involved in mechanisms of tubulointerstitial renal fibrosis and CKD progression. Its valuable predictive character is well associated with incident renal outcomes and improves risk prediction in incident CKD. Fibroblast growth factor-23 is an interesting biomarker for an expected decline of CDK and heart failure. FGF-23 is involved in the body's regulation of calcium-phosphate metabolism. FGF-23 level increases with decline of renal function. By an increase of renal phosphate excretion by FGF-23, circulating calcitriol levels are decreased, leading to secondary hyperparathyroidism (110, 111).

### Cardio-Hepatic Interaction

Impairment of cardiac function may result in hepatic failure and vice versa. HF induces liver hypoperfusion and hepatic congestion. In cirrhotic cardiomyopathy, proinflammatory conditions cause cardiac injury and a shift in myosin heavy chain subtype a to the weaker b isoform (112). Furthermore, liver-derived toxic factors cause circulatory abnormalities by arterial dilation and hyperdynamic circulation. Further progression of liver failure with concomitant arterial dilatation results in an exhausting of cardiac systolic reserve. Therefore, the heart is unable to further increase cardiac output and the resulting underfilling in the arterial system decreases the effective circulatory volume (113). In the CHARM trial, bilirubin was proven as a prognostic marker of worsening cardiovascular outcome and all-cause mortality. Similarly, increased transaminases, Gamma-glutamyl transferase (GGT) and alkaline phosphatase level (ALP) demonstrated increased mortality in patients with an advanced heart failure (114). Hypoalbuminemia is common in one in four heart failure patients, caused by systemic inflammation and a catabolic state along with hepatic impairment. In the Everest trial, low albumin was markedly associated with increased all-cause mortality and cardiovascular mortality, as well as frequency of re-hospitalization (115). Liver X receptors (LXRs), including LXRa and LXRb, are of great therapeutic interest because of their role as mediators of lipid and glucose metabolism, cholesterol homeostasis, and inflammation. Elevated cellular levels of cholesterol stimulate transcriptional activity of LXRs (116) and therefore, compensate reverse cholesterol transport, prevent diabetes-induced inflammation, and hamper pro-inflammatory macrophages (117). Effects of LXR on the cardiac system implement the decrease of cardiomyocyte hypertrophy, cardiomyocyte loss, and fibrotic remodeling. LXRs also promote angiogenesis within the myocardium and increase the capacity for glucose uptake and utilization (118). Altered LXR signaling pathways are associated with co-morbidities in heart failure, including atherosclerosis, hypertension, diabetes, obesity and chronic kidney disease (119).

### Cardiac-Gastrointestinal Interaction

Chronic heart failure induces a depletion of bacterial richness, in particular of butyrate-producing bacteria. Butyrate causes local anti-inflammatory effects in the intestinal mucosa and activates regulatory T cells (120). In patients with heart failure, the mucosal barrier is harmed by intestinal ischemia and thus, toxic, gut-derived metabolites may be released into systemic circulation (121). Trimethylamine (TMA) is an organic metabolite produced by gut microbiota. TMA is rapidly oxidized into trimethylamine N-oxide (TMAO) by flavin monooxygenase (FMO) enzymes in the liver and set free into the circulation. Although high TMAO levels are reported to be related with poor prognosis in HF, further studies evaluating the generation and metabolism of TMAO are needed (122). Gut dysbiosis has an impact on the progression of HF and chronic kidney disease. Therefore, prebiotics, probiotics and diet modification could be of prospective therapeutic benefit (123). The GutHeart study randomizes 150 patients with stable HF to receive either rifaximin, the probiotic yeast Saccharomyces boulardii, or no treatment (control group) for 3 months (124).

## “Out of the Box” Biomarkers

### Tumor Markers

Numerous studies evaluating tumor markers in heart failure have been conducted. To date, CA 125 seems to be the most promising. Several studies found a significant correlation between CA 125 and the different stages of heart failure in different populations, both in chronic and acute heart failure (125). The correlation was consistent throughout various underlying causes, such as hypertrophic cardiomyopathy (HCM) or mitral stenosis (126). Higher values of CA 125 were observed in patients with pleural or pericardial effusion (127). Furthermore, there was a significant relationship between CA 125 and NT-proBNP (128). CA 125 correlates with TNF-Alpha, IL-6 and IL-10 and therefore is the only tumor marker found to be closely related to the cytokine system (129). CA 125 could provide additional value in treatment guidance. Higher CA 125 levels in heart failure patients are predictive for more deaths and rehospitalizations (130) and a combination of BNP and CA 125 improves risk stratification at 6 months (131). Thus, the marker shows potential to influence medical therapy (132). Other tumor markers such as CEA, AFP, CA 15-3, CA 19-9, CA 724, NSE, and CYFRA 21-1 failed to provide promising results (133).

### Osteopontin

Myocardial expression of extracellular matrix osteopontin is associated with cardiac hypertrophy and is increased after development of heart failure (134, 135) in both ischemic and dilated cardiomyopathy (136). The expression of osteopontin increases with the severity of heart failure and seems to be a major regulator of myocardial remodeling where it potentiates galectin-3 up-regulation and secretion (137). After heart transplantation (HTX), osteopontin plasma levels decrease significantly, whereas those results were not observed in patients receiving left ventricular assist device (LVAD) support (138). Osteopontin improves diagnostic accuracy for acute congestive heart failure when combined with NT-proBNP, but is also an independent predictor of death and provides higher prognostic value of acute heart failure rehospitalizations than NT-proBNP (139). In a multi-marker analysis from 2017, only osteopontin and neuropilin predicted outcome in heart failure patients with preserved ejection fraction (HFpEF) (140).

### Neuropilin-1

Neuropilin-1 is a cell surface receptor that binds vascular endothelial growth factor and therefore serves as a marker of angiogenesis. In a multi-marker analysis in patients with HfpEF, Neuropilin served as only one of two biomarkers with independent prediction of outcome (141). *In vivo* studies in knockout mice suggest a cardio-protective role of neuropilin, as the animals developed higher rates of cardiomyopathy and heart failure compared to the controls (142).

### Endothelin-1

Plasma endothelin-1 is a powerful vasoconstrictor and has positive inotropic effects. It is produced by the vascular endothelium and cardiac myocytes and is elevated in congestive heart failure. The degree of plasma elevation correlates with the severity of heart failure (143). Administration of an endothelin-1 antagonist (Bosentan) reduces blood pressure, pulmonary artery pressure, pulmonary artery wedge pressure and right atrial pressure (144). Long-term treatment greatly improved the survival of rats with chronic heart failure (33). However, treatment in humans showed no beneficial effect as demonstrated in the ENABLE study, which included more than 1,600 patients over the follow-up of 9 months. Additionally, the treatment with Bosentan in HFpEF failed to show beneficial effects (145). Side effects, such as fluid retention was increased in the first 2–4 weeks in the Bosentan group (146).

### Circulating Endothelial Progenitor Cells With Angiopoetic Phenotypes

Endothelial progenitor cells (EPCs) originated from the bone marrow-derived cells have previously found as powerful endogenous contributor of vascular wall maintenance and the endothelium integrity (147). EPCs play a pivotal role in the vasculogenesis, neovascularization, supporting endothelial function, tissue procetion and vascular repair (148). Normally, pro-inflammatory cytokines and growth factors contribute to increase the number of EPCs with angiopoetic phenotypes in the circulation and thereby tissue reparation is supported. Numerous CV conditions, such as HF, as well as diabetes mellitus, chronic kidney disease were associated with EPCs dysfunction due to several causes, i.e., exhausting endothelial precursor pool, cytokine-induce apoptosis, epigenetic impact, and insulin resistance. Recent studies have shown that development of HF and occurrence of HF-related clinical outcomes were related to lowered number and weak function and survival of circulating EPCs (149, 150). Several phenotypes of EPCs (CD45dimCD34+, CD45dimCD34+CD133+, CD45dimCD34+CD133+VEGFR+,CD45dimCD34+CD133+Tei2+) have demonstrated additional prognostic information about mortality and HF-related admission regardless of serum levels of numerous biomarkers (NT-proBNP, galectin-3, hs-CRP, osteoprotegerin, osteopontin) and co-morbidities including diabetes mellitus, abdominal obesity and chronic renal disease (150, 151). Thus, it has been expected that they could possible utilize as target for the HF therapy (152).

There is a large number of evidence of the fact that ARBs, ARNI, mineralocorticoid antagonists have yielded remarkable impact on the clinical outcomes among HFrEF and HFpEF patients through prevention of adverse cardiac remodeling and improving endothelial function in close connection with an increase in the viability and the number of circulating EPCs (153, 154). Perhaps, continuous monitoring for EPCs function and number could be promising biomarker-guided therapy for HF patients with respect to daily dose adjustment of the drugs. Although these findings appear to be intriguing, the molecular mechanisms underlying improvement of HF outcomes intimately related to attenuation of EPCs dysfunction is unclear and whether targeting EPCs count and function is a feasible strategy for ameliorating HF development and progression remains uncertain, while it is promising.

## Conclusion

Multi-marker analysis can provide valuable information about the predominant processes in the complex, multifunctional and individual pathophysiology of heart failure. Biomarker-based therapy has the potential for providing prospective heart failure patients with tailored treatments adapted to the multi-faceted aspects of heart failure. Introduction of a new multi-marker-based therapeutic approach and resulting therapies as currently being investigated in clinical studies may offer new disease management options to potentially decrease the morbidity and mortality of HF patients.

## Limitations

The lack of cardiac specifity is of major limitation. The variability of serum biomarkers is influenced by age, sex, co – morbidities and renal failure. Therefore, multimarker panels are recommended to improve prognostic utility.

Intraindividual variation of serum marker is of further diagnostic limitation (155). Environmental circumstances, including height, are reported to have an influence on sST2, suPAR, H-FABP and GDF-15 levels (156). Furthermore, intensive physical exercise is reported to influence cardiac biomarkers (157, 158).

## Author Contributions

AT, MM, BO, PJ, DF, MH, and AB performed literature research and prepared the manuscript. KK, LM, UH, AB, and ML revised the manuscript. All authors contributed to the article and approved the submitted version.

## Conflict of Interest

The authors declare that the research was conducted in the absence of any commercial or financial relationships that could be construed as a potential conflict of interest.

## References

[1] Xie W Zheng F Song X Zhong B Yan L Renin-angiotensin-aldosterone system blockers for heart failure with reduced ejection fraction or left ventricular dysfunction: network meta-analysis. Int J Cardiol. (2016) 205:65–71. 10.1016/j.ijcard.2015.12.010 26720043

[2] Goldenberg I Huang D Nielsen J. The role of implantable cardioverter-defibrillators and sudden cardiac death prevention: indications, device selection, and outcome. Eur Heart J. (2020) 41:2003–11. 10.1093/eurheartj/ehz788 31713598

[3] Ponikowski P Voors A Anker S Bueno H Cleland JGF Coats AJS ESC Guidelines for the diagnosis and treatment of acute and chronic heart failure: the Task Force for the diagnosis and treatment of acute and chronic heart failure of the European Society of Cardiology (ESC). Developed with the special contribution of the Heart Failure Association (HFA) of the ESC. Eur Heart J. (2016) 37:2129–200. 10.1093/eurheartj/ehw128 27206819

[4] Samsky MD Patel CB DeWald T Smith AD Felker GM Rogers JG Hernandez AF. Cardiohepatic interactions in heart failure. J Am Coll Cardiol. (2013) 61:2397–405. 10.1016/j.jacc.2013.03.042 23603231

[5] Velagaleti RS Gona P Larson MG Wang TJ Levy D Benjamin EJ. Multimarker approach for the prediction of heart failure incidence in the community. Circulation. (2010) 122:1700–6. 10.1161/CIRCULATIONAHA.109.929661 20937976 PMC2993157

[6] Vuolteenaho O Ala-Kopsala M Ruskoaho H. BNP as a biomarker in heart disease. Adv Clin Chem. (2005) 40:1–36. 10.1016/S0065-2423(05)40001-3 16355919

[7] Allen LA Felker GM. Multi-marker strategies in heart failure: clinical and statistical approaches. Heart Fail Rev. (2010) 15:343–9. 10.1007/s10741-009-9144-z 19412735 PMC3961582

[8] Deswal A Cytokines and cytokine receptors in advanced heart failure : an analysis of the cytokine database from the vesnarinone trial (VEST). Circulation. (2001) 103:2055–9. 10.1161/01.CIR.103.16.2055 11319194

[9] Savarese G. Global public health burden of heart failure. Card Fail Rev. (2017) 3:7–11. 10.15420/cfr.2016:25:2 28785469 PMC5494150

[10] Dutka M Bobiński R Ulman-Włodarz I Hajduga M Bujok J Pajak C. Various aspects of inflammation in heart failure. Heart Fail Rev. (2020) 25:537–48. 10.1007/s10741-019-09875-1 31705352 PMC7181445

[11] Pellicori P Zhang J Cuthbert J Urbinati A Kazmi S Clark AL. High-sensitivity C-reactive protein in chronic heart failure: patient characteristics, phenotypes, and mode of death. Cardiovasc Res. (2020) 116:91–100. 10.1093/cvr/cvz198 31350553

[12] Ikonomidis I Lekakis JP Nikolaou M Paraskevaidis I Andreadou I Kaplanoglou T. Inhibition of interleukin-1 by anakinra improves vascular and left ventricular function in patients with rheumatoid arthritis. Circulation. (2008) 117:2662–9. 10.1161/CIRCULATIONAHA.107.731877 18474811

[13] Kalogeropoulos A Georgiopoulou V Psaty BM Rodondi N Smith AL Harrison DG. Inflammatory markers and incident heart failure risk in older adults. The health, aging and body composition. J Am Coll Cardiol. (2010) 55:2129–37. 10.1016/j.jacc.2009.12.045 20447537 PMC3267799

[14] Szekely Y Arbel Y. A review of interleukin-1 in heart disease: where do we stand today? Cardiol Ther. (2018) 7:25–44. 10.1007/s40119-018-0104-3 29417406 PMC5986669

[15] Aleksova A Beltrami AP Carriere C Barbati G Lesizza P Perrieri-Montanino M. Interleukin-1β levels predict long-term mortality and need for heart transplantation in ambulatory patients affected by idiopathic dilated cardiomyopathy. Oncotarget. (2017) 8:25131–40. 10.18632/oncotarget.15349 28212578 PMC5421915

[16] Van Tassell BW Seropian IM Toldo S Mezzaroma E Abbate A. Interleukin-1beta induces a reversible cardiomyopathy in the mouse. Inflamm Res. (2013) 62:637–40. 10.1007/s00011-013-0625-0 23649041

[17] Radin MJ Holycross BJ Dumitrescu C Kelley R Altschuld RA. Leptin modulates the negative inotropic effect of interleukin-1beta in cardiac myocytes. Mol Cell Biochem. (2008) 315:179–84. 10.1007/s11010-008-9805-6 18535786

[18] Pascual-Figal DA Bayes-Genis A Asensio-Lopez MC Hernandez-Vicente A Garrido-Bravo I Pastor-Perez F. The interleukin-1 axis and risk of death in patients with acutely decompensated heart failure. J Am Coll Cardiol. (2019) 73:1016–25. 10.1016/j.jacc.2018.11.054 30846095

[19] Felker GM Anstrom KJ Adams KF Ezekowitz JA Fiuzat M Houston-Miller N Effect of natriuretic peptide-guided therapy on hospitalization or cardiovascular mortality in high-risk patients with heart failure and reduced ejection fraction: a randomized clinical trial. JAMA. (2017) 318:713–20. 10.1001/jama.2017.10565 28829876 PMC5605776

[20] Van Tassell BW Arena RA Toldo S Mezzaroma E Azam T Seropian IM. Enhanced interleukin-1 activity contributes to exercise intolerance in patients with systolic heart failure. PLoS ONE. (2012) 7:e33438. 10.1371/journal.pone.0033438 22438931 PMC3306393

[21] Van Tassell BW Abouzaki NA Oddi Erdle C Carbone S Trankle CR Melchior RD. Interleukin-1 blockade in acute decompensated heart failure: a randomized, double-blinded, placebo-controlled pilot study. J Cardiovasc Pharmacol. (2016) 67:544–51. 10.1097/FJC.0000000000000378 26906034 PMC5749643

[22] Van Tassell BW Canada J Carbone S Trankle C Buckley L Oddi Erdle C. Interleukin-1 blockade in recently decompensated systolic heart failure: results from REDHART (Recently Decompensated Heart Failure Anakinra Response Trial). Circ Heart Fail. (2017) 10:e004373. 10.1161/CIRCHEARTFAILURE.117.004373 29141858 PMC5699505

[23] Van Tassell BW Buckley LF Carbone S Trankle CR Canada JM Dixon DL. Interleukin-1 blockade in heart failure with preserved ejection fraction: rationale and design of the Diastolic Heart Failure Anakinra Response Trial 2 (D-HART2). Clin Cardiol. (2017) 40:626–32. 10.1002/clc.22719 28475816 PMC5744484

[24] Everett BM Cornel JH Lainscak M Anker SD Abbate A Thuren T. Anti-inflammatory therapy with canakinumab for the prevention of hospitalization for heart failure. Circulation. (2019) 139:1289–99. 10.1161/CIRCULATIONAHA.118.038010 30586730

[25] Deswal A Petersen NJ Feldman AM Young JB White BG Mann DL. Cytokines and cytokine receptors in advanced heart failure: an analysis of the cytokine database from the Vesnarinone trial (VEST). Circulation. (2001) 103:2055–9. 10.1161/01.cir.103.16.2055 11319194

[26] Sun M Dawood F Wen WH Chen M Dixon I Kirshenbaum LA. Excessive tumor necrosis factor activation after infarction contributes to susceptibility of myocardial rupture and left ventricular dysfunction. Circulation. (2004) 110:3221–8. 10.1161/01.CIR.0000147233.10318.23 15533863

[27] Mann DL McMurray JJ Packer M Swedberg K Borer JS Colucci WS. Targeted anticytokine therapy in patients with chronic heart failure: results of the Randomized Etanercept Worldwide Evaluation (RENEWAL). Circulation. (2004) 109:1594–602. 10.1161/01.CIR.0000124490.27666.B2 15023878

[28] Chung ES Packer M Lo KH Fasanmade AA Willerson JT. Anti TNFTACHFI. Randomized, double-blind, placebo-controlled, pilot trial of infliximab, a chimeric monoclonal antibody to tumor necrosis factor-alpha, in patients with moderate-to-severe heart failure: results of the anti-TNF Therapy Against Congestive Heart Failure (ATTACH) trial. Circulation. (2003) 107:3133–40. 10.1161/01.CIR.0000077913.60364.D2 12796126

[29] Damas JK Gullestad L Aukrust P. Cytokines as new treatment targets in chronic heart failure. Curr Control Trials Cardiovasc Med. (2001) 2:271–7. 10.1186/CVM-2-6-271 11806813 PMC64832

[30] Nakamura M Sadoshima J. Mechanisms of physiological and pathological cardiac hypertrophy. Nat Rev Cardiol. (2018) 15:387–407. 10.1038/s41569-018-0007-y 29674714

[31] Li L Zhao Q Kong W. Extracellular matrix remodeling and cardiac fibrosis. Matrix Biol. (2018) 69:490–506. 10.1016/j.matbio.2018.01.013 29371055

[32] Azevedo PS Polegato BF Minicucci MF Paiva SA Zornoff LA. Cardiac remodeling: concepts, clinical impact, pathophysiological mechanisms and pharmacologic treatment. Arq Bras Cardiol. (2016) 106:62–9. 10.5935/abc.20160005 26647721 PMC4728597

[33] Gibb AA Hill BG. Metabolic coordination of physiological and pathological cardiac remodeling. Circ Res. (2018) 123:107–28. 10.1161/CIRCRESAHA.118.312017 29929976 PMC6023588

[34] Talman V Ruskoaho H. Cardiac fibrosis in myocardial infarction-from repair and remodeling to regeneration. Cell Tissue Res. (2016) 365:563–81. 10.1007/s00441-016-2431-9 27324127 PMC5010608

[35] Packer M. Leptin-aldosterone-neprilysin axis: identification of its distinctive role in the pathogenesis of the three phenotypes of heart failure in people with obesity. Circulation. (2018) 137:1614–31. 10.1161/CIRCULATIONAHA.117.032474 29632154

[36] Dieplinger B Mueller T. Soluble ST2 in heart failure. Clin Chim Acta. (2015) 443:57–70. 10.1016/j.cca.2014.09.021 25269091

[37] Maisel AS Richards AM Pascual-Figal D Mueller C. Serial ST2 testing in hospitalized patients with acute heart failure. Am J Cardiol. (2015) 115:23. 10.1016/j.amjcard.2015.01.038 25682437

[38] Kakkar R Lee RT. The IL-33/ST2 pathway: therapeutic target and novel biomarker. Nat Rev Drug Discov. (2008) 7:827–40. 10.1038/nrd2660 18827826 PMC4277436

[39] Griesenauer B Paczesny S. The ST2/IL-33 Axis in immune cells during inflammatory diseases. Front Immunol. (2017) 8:475. 10.3389/fimmu.2017.00475 28484466 PMC5402045

[40] Aimo A Januzzi JLJr Bayes-Genis A Vergaro G Sciarrone P Passino C. Clinical and prognostic significance of sST2 in heart failure: JACC review topic of the week. J Am Coll Cardiol. (2019) 74:2193–203. 10.1016/j.jacc.2019.08.1039 31648713

[41] Aimo A Vergaro G Passino C Ripoli A Ky B Miller WL. Prognostic value of soluble suppression of tumorigenicity-2 in chronic heart failure: a meta-analysis. JACC Heart Fail. (2017) 5:280–6. 10.1016/j.jchf.2016.09.010 27816512

[42] Zagidullin N Motloch LJ Gareeva D Hamitova A Lakman I Krioni I. Combining novel biomarkers for risk stratification of two-year cardiovascular mortality in patients with ST-elevation myocardial infarction. J Clin Med. (2020) 9:550. 10.3390/jcm9020550 32085400 PMC7073894

[43] Yu J Oh PC Kim M Moon J Park YM Lee K. Improved early risk stratification of patients with ST-segment elevation myocardial infarction undergoing primary percutaneous coronary intervention using a combination of serum soluble ST2 and NT-proBNP. PLoS ONE. (2017) 12:e0182829. 10.1371/journal.pone.0182829 28796845 PMC5552027

[44] Jenkins WS Roger VL Jaffe AS Weston SA AbouEzzeddine OF Jiang R. Prognostic value of soluble ST2 after myocardial infarction: a community perspective. Am J Med. (2017) 130:23. 10.1016/j.amjmed.2017.02.034 28344136 PMC5572469

[45] Jirak P Mirna M Wernly B Paar V Thieme M Betge S. Analysis of novel cardiovascular biomarkers in patients with peripheral artery disease (PAD). Miner Med. (2018) 109:443–50. 10.23736/S0026-4806.18.05628-8 29652038

[46] Mirna M Rohm I Jirak P Wernly B Baz L Paar V. Analysis of novel cardiovascular biomarkers in patients with pulmonary hypertension (PH). Heart Lung Circ. (2020) 29:337–44. 10.1016/j.hlc.2019.03.004 31327702

[47] Adela R Banerjee SK. GDF-15 as a target and biomarker for diabetes and cardiovascular diseases: a translational prospective. J Diabetes Res. (2015) 2015:490842. 10.1155/2015/490842 26273671 PMC4530250

[48] Wollert KC Kempf T Wallentin L. Growth differentiation factor 15 as a biomarker in cardiovascular disease. Clin Chem. (2017) 63:140–51. 10.1373/clinchem.2016.255174 28062617

[49] Farhan S Freynhofer MK Brozovic I Bruno V Vogel B Tentzeris I. Determinants of growth differentiation factor 15 in patients with stable and acute coronary artery disease. A prospective observational study. Cardiovasc Diabetol. (2016) 15:60. 10.1186/s12933-016-0375-8 27056183 PMC4825089

[50] Berezin AE. Diabetes mellitus related biomarker: the predictive role of growth-differentiation factor-15. Diabetes Metab Syndr. (2016) 10:9. 10.1016/j.dsx.2015.09.016 26482961

[51] Savic-Radojevic A Pljesa-Ercegovac M Matic M Simic D Radovanovic S Simic T. Novel biomarkers of heart failure. Adv Clin Chem. (2017) 79:93–152. 10.1016/bs.acc.2016.09.002 28212715

[52] Tong L Deli S Jianzeng D Pinghui Z Jie L Wei L. Current understanding of the pathophysiology of myocardial fibrosis and its quantitative assessment in heart failure. Front Physiol. (2017) 8:238. 10.3389/fphys.2017.00238 28484397 PMC5402617

[53] Humeres C Frangogiannis NG. Fibroblasts in the infarcted, remodeling, and failing heart. JACC Basic Transl Sci. (2019) 4:449–67. 10.1016/j.jacbts.2019.02.006 31312768 PMC6610002

[54] Wynn TA Ramalingam TR. Mechanisms of fibrosis: therapeutic translation for fibrotic disease. Nat Med. (2012) 18:1028–40. 10.1038/nm.2807 22772564 PMC3405917

[55] Bing R Dweck MR. Myocardial fibrosis: why image, how to image and clinical implications. Heart. (2019) 105:1832–40. 10.1136/heartjnl-2019-315560 31649047 PMC6900237

[56] Zhen-Guo M Cardiac fibrosis: new insights into the pathogenesis. Int J Biol Sci. 14:1645–57. 10.7150/ijbs.28103 PMC6216032 30416379

[57] Tzavlaki K Moustakas A. TGF-β Signaling. Biomolecules. (2020) 10:487. 10.3390/biom10030487 32210029 PMC7175140

[58] Działo E Tkacz K Błyszczuk P. Crosstalk between the TGF-β and WNT signalling pathways during cardiac fibrogenesis. Acta Biochim Pol. (2018) 65:341–9. 10.18388/abp.2018_2635 30040870

[59] Parichatikanond W Luangmonkong T Mangmool S Kurose H. Therapeutic targets for the treatment of cardiac fibrosis and cancer: focusing on TGF-β signaling. Front Cardiovasc Med. (2020) 7:34. 10.3389/fcvm.2020.00034 32211422 PMC7075814

[60] Ma ZG Yuan YP Wu HM Zhang X Tang QZ. Cardiac fibrosis: new insights into the pathogenesis. Int J Biol Sci. (2018) 14:1645–57. 10.7150/ijbs.28103 30416379 PMC6216032

[61] Travers JG Kamal FA Robbins J Yutzey KE Blaxall BC. Cardiac fibrosis: the fibroblast awakens. Circ Res. (2016) 118:1021–40. 10.1161/CIRCRESAHA.115.306565 26987915 PMC4800485

[62] Li T Jiang S Ni B Cui Q Liu Q Zhao H. Discontinued drugs for the treatment of cardiovascular disease from 2016 to 2018. Int J Mol Sci. (2019) 20:4513. 10.3390/ijms20184513 31547243 PMC6769515

[63] Silverman MG Glaser R Cavender MA Hamershock R O'Donoghue ML Sabatine MS Abstract 19771: impact of losmapimod on cardiovascular death and heart failure after ST-elevation myocardial infarction in LATITUDE TIMI-60. Circulation. 134:A19771.

[64] Münzel T Camici GG Maack C Bonetti NR Fuster V Jason C. Impact of oxidative stress on the heart and vasculature part 2 of a 3-part series. J Am Coll Cardiol. (2017) 70:212–29. 10.1016/j.jacc.2017.05.035 28683969 PMC5663297

[65] Schirone L Forte M Palmerio S Yee D Nocella C Angelini R. A review of the molecular mechanisms underlying the development and progression of cardiac remodeling. Oxid Med Cell Longev. (2017) 2017:3920195. 10.1155/2017/3920195 28751931 PMC5511646

[66] Sato Y Fujiwara H Takatsu Y. Cardiac troponin and heart failure in the era of high-sensitivity assays. J Cardiol. (2012) 60:160–7. 10.1016/j.jjcc.2012.06.007 22867801

[67] Eiserich JP Hristova M Cross CE Jones AD Freeman BA Halliwell B. Formation of nitric oxide-derived inflammatory oxidants by myeloperoxidase in neutrophils. Nature. (1998) 391:393–7. 10.1038/34923 9450756

[68] Ali M Pulli B Courties G Tricot B Sebas M Iwamoto Y. Myeloperoxidase inhibition improves ventricular function and remodeling after experimental myocardial infarction. JACC Basic Transl Sci. (2016) 1:633–43. 10.1016/j.jacbts.2016.09.004 30167547 PMC6113523

[69] Tang WH Brennan ML Philip K Tong W Mann S Van Lente F. Plasma myeloperoxidase levels in patients with chronic heart failure. Am J Cardiol. (2006) 98:796–9. 10.1016/j.amjcard.2006.04.018 16950188

[70] Ng LL Pathik B Loke IW Squire IB Davies JE. Myeloperoxidase and C-reactive protein augment the specificity of B-type natriuretic peptide in community screening for systolic heart failure. Am Heart J. (2006) 152:94–101. 10.1016/j.ahj.2005.09.020 16824837

[71] Reichlin T Socrates T Egli P Potocki M Breidthardt T Arenja N. Use of myeloperoxidase for risk stratification in acute heart failure. Clin Chem. (2010) 56:944–51. 10.1373/clinchem.2009.142257 20413430

[72] Michowitz Y Kisil S Guzner-Gur H Rubinstein A Wexler D Sheps D. Usefulness of serum myeloperoxidase in prediction of mortality in patients with severe heart failure. Isr Med Assoc J. (2008) 10:884–8. 19160948

[73] Pacher P Nivorozhkin A Szabo C. Therapeutic effects of xanthine oxidase inhibitors: renaissance half a century after the discovery of allopurinol. Pharmacol Rev. (2006) 58:87–114. 10.1124/pr.58.1.6 16507884 PMC2233605

[74] Santos CX Anjos EI Augusto O. Uric acid oxidation by peroxynitrite: multiple reactions, free radical formation, and amplification of lipid oxidation. Arch Biochem Biophys. (1999) 372:285–94. 10.1006/abbi.1999.1491 10600166

[75] Culleton BF Larson MG Kannel WB Levy D. Serum uric acid and risk for cardiovascular disease and death: the Framingham Heart Study. Ann Intern Med. (1999) 131:7–13. 10.7326/0003-4819-131-1-199907060-00003 10391820

[76] Zhang J Dierckx R Mohee K Clark AL Cleland JG. Xanthine oxidase inhibition for the treatment of cardiovascular disease: an updated systematic review and meta-analysis. ESC Heart Fail. (2017) 4:40–5. 10.1002/ehf2.12112 28217311 PMC5292634

[77] Filippatos GS Ahmed MI Gladden JD Mujib M Aban IB Love TE. Hyperuricaemia, chronic kidney disease, and outcomes in heart failure: potential mechanistic insights from epidemiological data. Eur Heart J. (2011) 32:712–20. 10.1093/eurheartj/ehq473 21199831 PMC3056205

[78] Vaduganathan M Greene SJ Ambrosy AP Mentz RJ Subacius HP Chioncel O. Relation of serum uric acid levels and outcomes among patients hospitalized for worsening heart failure with reduced ejection fraction (from the efficacy of vasopressin antagonism in heart failure outcome study with tolvaptan trial). Am J Cardiol. (2014) 114:1713–21. 10.1016/j.amjcard.2014.09.008 25312638

[79] Borghi C Cosentino E Bragagni A. Hyperuricemia and mortality in heart failure: Is it time to change the route? Eur J Intern Med. (2020) 72:40–1. 10.1016/j.ejim.2020.01.005 31980331

[80] Vardi M Levy NS Levy AP. Vitamin E in the prevention of cardiovascular disease: the importance of proper patient selection. J Lipid Res. (2013) 54:2307–14. 10.1194/jlr.R026641 23505320 PMC3735930

[81] Pasupathy S Tavella R Grover S Raman B Procter YT Mahadavan G. Early use of N-acetylcysteine with nitrate therapy in patients undergoing primary percutaneous coronary intervention for ST-segment-elevation myocardial infarction reduces myocardial infarct size (the NACIAM Trial [N-acetylcysteine in acute myocardial infarction]). Circulation. (2017) 136:894–903. 10.1161/CIRCULATIONAHA.117.027575 28634219

[82] Van der Pol A van Gilst WH Voors AA van der Meer P. Treating oxidative stress in heart failure: past, present and future. Eur J Heart Fail. (2019) 21:425–35. 10.1002/ejhf.1320 30338885 PMC6607515

[83] Lee RC Feinbaum RL Ambros V. The C. elegans heterochronic gene lin-4 encodes small RNAs with antisense complementarity to lin-14. Cell. (1993) 75:843–54. 10.1016/0092-8674(93)90529-Y 8252621

[84] Mohr AM Mott JL. Overview of microRNA biology. Semin Liver Dis. (2015) 35:3–11. 10.1055/s-0034-1397344 25632930 PMC4797991

[85] Kim S Song ML Min H Hwang I Kyu S Taeg B. MiRNA biogenesis-associated RNase III nucleases drosha and dicer are upregulated in colorectal adenocarcinoma. Oncol Lett. (2017) 14:4379–83. 10.3892/ol.2017.6674 28943952 PMC5605964

[86] Mirna M Para V Rezar R Topf A Eber M Hoppe U. MicroRNAs in inflammatory heart diseases and sepsis-induced cardiac dysfunction: a potential scope for the future? Cells. (2019) 8:1352. 10.3390/cells8111352 31671621 PMC6912436

[87] Wang H Cai J The role of microRNAs in heart failure. Biochim Biophys Acta Mol Basis Dis. (2019) 1863:2019–30. 10.1016/j.bbadis.2016.11.034 27916680

[88] Carè A Catalucci D Felicetti F Bonci D Addario A Gallo P. MicroRNA-133 controls cardiac hypertrophy. Nat Med. (2007) 13:613–8. 10.1038/nm1582 17468766

[89] Wang J Yang X The function of miRNA in cardiac hypertrophy. Cell Mol Life Sci. (2012) 69:3561–70. 10.1007/s00018-012-1126-y 22926414 PMC3474911

[90] Melak T Baynes HW. Circulating microRNAs as possible biomarkers for coronary artery disease: a narrative review. EJIFCC. (2019) 30:179–94. 31263392 PMC6599194

[91] Ultimo S Zauli G Martelli AM Vitale M McCubrey JA Capitani S. Cardiovascular disease-related miRNAs expression: potential role as biomarkers and effects of training exercise. Oncotarget. (2018) 9:17238–54. 10.18632/oncotarget.24428 29682219 PMC5908320

[92] Jaguszewski M Osipova J Ghadri JR Napp LC Widera C Franke J. A signature of circulating microRNAs differentiates takotsubo cardiomyopathy from acute myocardial infarction. Eur Heart J. (2014) 35:999–1006. 10.1093/eurheartj/eht392 24046434 PMC3985061

[93] Seronde MF Vausort M Gayat E Goretti E Ng LL Squire IB. Circulating microRNAs and outcome in patients with acute heart failure. PLoS ONE. (2015) 10:e0142237. 10.1371/journal.pone.0142237 26580972 PMC4651494

[94] Vegter EL Ovchinnikova ES van Veldhuisen DJ Jaarsma T Berezikov E van der Meer P. Low circulating microRNA levels in heart failure patients are associated with atherosclerotic disease and cardiovascular-related rehospitalizations. Clin Res Cardiol. (2017) 106:598–609. 10.1007/s00392-017-1096-z 28293796 PMC5529487

[95] Wong LL Wang J Liew OW Richards AM Chen YT MicroRNA and heart failure. Int J Mol Sci. (2016) 17:502 10.3390/ijms17040502 27058529 PMC4848958

[96] Ovchinnikova ES Schmitter D Vegter EL ter Maaten JM Valente MA Liu LC. Signature of circulating microRNAs in patients with acute heart failure. Eur J Heart Fail. (2016) 18:414–23. 10.1002/ejhf.332 26345695

[97] Watson CJ Gupta SK O'Connell E Thum S Glezeva N Fendrich J. MicroRNA signatures differentiate preserved from reduced ejection fraction heart failure. Eur J Heart Fail. (2015) 17:405–15. 10.1002/ejhf.244 25739750 PMC4418397

[98] Salvatore De Rosa F Eposito C Carella A Strangio G Ammirati J Sabatino FG. Transcoronary concentration gradients of circulating microRNAs in heart failure. Eur J Heart Fail. (2018) 20:920–7. 10.1002/ejhf.1119 29314582

[99] Roncarati R Viviani Anselmi C Losi MA Papa L Cavarretta E Martins P. Circulating miR-29a, among other up-regulated microRNAs, is the only biomarker for both hypertrophy and fibrosis in patients with hypertrophic cardiomyopathy. J Am Coll Cardiol. (2014) 63:920–7. 10.1016/j.jacc.2013.09.041 24161319

[100] Derda AA Thum S Lorenzen JM Bavendiek U Heineke J Keyser B. Blood-based microRNA signatures differentiate various forms of cardiac hypertrophy. Int J Cardiol. (2015) 196:115–22. 10.1016/j.ijcard.2015.05.185 26086795 PMC4936391

[101] Janssen HLA Reesink HW Lawitz EJ Zeuzem S Rodriguez-Torres M Patel K. Treatment of HCV infection by targeting microRNA. N Engl J Med. (2013) 368:1685–94. 10.1056/NEJMoa1209026 23534542

[102] Nassar D Blanpain C. Cancer stem cells: basic concepts and therapeutic implications. Annu Rev Pathol Mech Dis. (2016) 11:47–76. 10.1146/annurev-pathol-012615-044438 27193450

[103] Thum T Gross C Fiedler J Fischer T Kissler S Bussen M. MicroRNA-21 contributes to myocardial disease by stimulating MAP kinase signalling in fibroblasts. Nature. (2008) 456:980–4. 10.1038/nature07511 19043405

[104] Roy S Khanna S Hussain S Al E. MicroRNA expression in response to murine myocardial infarction: miR-21 regulates fibroblast metalloprotease-2 via phosphatase and tensin homologue. Cardiovasc Res. (2020) 82:21–9. 10.1093/cvr/cvp015 19147652 PMC2652741

[105] Lorenzen JM Schauerte C Hübner A Kölling M Martino F Scherf K. Osteopontin is indispensible for AP1-mediated angiotensin II-related miR-21 transcription during cardiac fibrosis. Eur Heart J. (2015) 36:2184–96. 10.1093/eurheartj/ehv109 25898844 PMC4543785

[106] Liu YL Wu W Xue Y Gao M Yan Y Kong Q. MicroRNA-21 and−146b are involved in the pathogenesis of murine viral myocarditis by regulating TH-17 differentiation. Arch Virol. (2013) 158:1953–63. 10.1007/s00705-013-1695-6 23588407

[107] Zhang Y Huang XR Wie LH Chung AC Yu CM Lan HY. MiR-29b as a therapeutic agent for angiotensin ii-induced cardiac fibrosis by targeting TGF-β/Smad3 signaling. Mol Ther. (2014) 22:974–85. 10.1038/mt.2014.25 24569834 PMC4015231

[108] Vegter EL Van Der Meer P De Windt LJ Pinto YM Voors AA MicroRNAs in heart failure: from biomarker to target for therapy. Eur J Heart Fail. (2016) 18:457–68. 10.1002/ejhf.495 26869172

[109] Tan K Sethi SK. Biomarkers in cardiorenal syndromes. Transl Biomark. (2014) 164:122–34. 10.1016/j.trsl.2014.04.011 24831739

[110] Niizuma S Iwanaga Y Yahata T Miyazaki S. Renocardiovascular biomarkers: from the perspective of managing chronic kidney disease and cardiovascular disease. Front Cardiovasc Med. (2017) 4:10. 10.3389/fcvm.2017.00010 28321399 PMC5337832

[111] Cediel G Santiago-Vacas E Bayes-Genis A Biomarkers and heart–kidney interaction. Eur Heart J Suppl. (2018) 20 (suppl_G):G28–36. 10.1093/eurheartj/suy021

[112] Ruiz-del-Árbol L Serradilla R. Cirrhotic cardiomyopathy. World J Gastroenterol. (2015) 21:11502–21. 10.3748/wjg.v21.i41.11502 26556983 PMC4631957

[113] Wiese S Hove JD Bendtsen F Møller S. Cirrhotic cardiomyopathy: pathogenesis and clinical relevance. Nat Rev Gastroenterol Hepatol. (2014) 11:177–86. 10.1038/nrgastro.2013.210 24217347

[114] Ambrosy AP Pang PS Khan S Konstam MA Fonarow GC Traver B. Clinical course and predictive value of congestion during hospitalization in patients admitted for worsening signs and symptoms of heart failure with reduced ejection fraction: findings from the EVEREST trial. Eur Heart J. (2013) 34:835–43. 10.1093/eurheartj/ehs444 23293303

[115] Mecklai A Subačius HM Konstam A Gheorghia M Butler J Ambrosy AP. In-hospital diuretic agent use and post-discharge clinical outcomes in patients hospitalized for worsening heart failure: insights from the EVEREST trial. JACC Heart Fail. (2016) 4:580–8. 10.1016/j.jchf.2016.02.008 27039131 PMC4930424

[116] Schulman IG. Liver X receptors link lipid metabolism and inflammation. FEBS Lett. (2017) 591:2978–91. 10.1002/1873-3468.12702 28555747 PMC5638683

[117] Tontonoz P. Liver X receptors in lipid signalling and membrane homeostasis. Nat Rev Endocrinol. (2018) 14:452–63. 10.1038/s41574-018-0037-x 29904174 PMC6433546

[118] Cannon MC van Gilst WH de Boer RA. Emerging role of liver X receptors in cardiac pathophysiology and heart failure. Basic Res Cardiol. (2016) 111:3. 10.1007/s00395-015-0520-7 26611207 PMC4661180

[119] Xanthopoulos A Randall S Kitai T Triposkiadis F. Heart failure and liver disease: cardiohepatic interactions. JACC Heart Fail. (2019) 7:87–97. 10.1016/j.jchf.2018.10.007 30553904

[120] Qiujin J Li H Zhou H Zhang X Zhang A Xie Y. Role and effective therapeutic target of gut microbiota in heart failure. Cardiovasc Ther. (2019) 2019:5164298. 10.1155/2019/5164298 31819762 PMC6885196

[121] Kummen M. Gut microbiota signature in heart failure defined from profiling of 2 independent cohorts. J Am Coll Cardiol. (2017) 71:1184–6. 10.1016/j.jacc.2017.12.057 29519360

[122] Fennema D Phillips IR Shephard EA. Trimethylamine and trimethylamine N-oxide, a flavin-containing monooxygenase 3 (FMO3)-mediated host-microbiome metabolic axis implicated in health and disease. Drug Metab Dispos. (2016) 44:1839–50. 10.1124/dmd.116.070615 27190056 PMC5074467

[123] Wilson TWH Kitai T Hazen SL. Gut microbiota in cardiovascular health and disease. Circ Res. (2017) 120:1183–96. 10.1161/CIRCRESAHA.117.309715 28360349 PMC5390330

[124] Mayerhofer CK Awoyemi AO Moscavitch SD Lappegård KT Hov JR Aukrust P. Design of the GutHeart—targeting gut microbiota to treat heart failure—trial: a Phase II, randomized clinical trial. ESC Heart Fail. (2018) 5:977–84. 10.1002/ehf2.12332 30088346 PMC6165929

[125] D'Aloia A Faggiano P Aurigemma G Bontempi L Ruggeri G Metra M. Serum levels of carbohydrate antigen 125 in patients with chronic heart failure: relation to clinical severity, hemodynamic and doppler echocardiographic abnormalities, and short-term prognosis. J Am Coll Cardiol. (2003) 41:1805–11. 10.1016/S0735-1097(03)00311-5 12767668

[126] Duman C Ercan E Tengiz I Bozdemir H Ercan EH Nalbantgil I. Elevated serum CA 125 levels in mitral stenotic patients with heart failure. Cardiology. (2003) 100:7–10. 10.1159/000072385 12975539

[127] Kouris N Zacharos I Kontogianni D Goranitou GS Sifaki MD Grassos HE. The significance of CA125 levels in patients with chronic congestive heart failure. Correlation with clinical and echocardiographic parameters. Eur J Heart Fail. (2005) 7:199–203. 10.1016/j.ejheart.2004.07.015 15701467

[128] Nunez J Sanchis J Bodi V Fonarow GC Núñez E Bertomeu-González V. Improvement in risk stratification with the combination of the tumour marker antigen carbohydrate 125 and brain natriuretic peptide in patients with acute heart failure. Eur Heart J. (2010) 31:1752–63. 10.1093/eurheartj/ehq142 20501480

[129] Ma J Zhao Y Wang Y Wang Y Guo Y Li J. Tumor marker levels in patients aged 85 years and older with chronic heart failure. Eur J Int Med. (2013) 24:440–3. 10.1016/j.ejim.2013.04.002 23643288

[130] Durak A Resic N Kulic M Pecar E Dzubur A Dilic M. Serum level of tumor marker carbohydrate antigen-CA125 in heart failure. Med Arh. (2013) 67:241–4. 10.5455/medarh.2013.67.241-244 24520743

[131] Varol E ·Ozaydin M Altinbas A Aslan SM Dogan A Dede O. Elevated carbohydrate antigen 125 levels in hypertrophic cardiomyopathy patients with heart failure. Heart Vessels. (2007) 22:30–3. 10.1007/s00380-006-0938-9 17285443

[132] Kosar F Aksoy Y Ozguntekin G Ozerol I Varol E. Relationship between cytokines and tumour markers in patients with chronic heart failure. Eur J Heart Fail. (2006) 8:270–4. 10.1016/j.ejheart.2005.09.002 16309955

[133] Faggiano P D'Aloia A Brentana L. Serum levels of different tumour markers in patients with chronic heart failure. Eur J Heart Fail. (2005) 7:57–61. 10.1016/j.ejheart.2004.04.009 15642532

[134] Stawowy P Blaschke F Pfautsch P Goetze S Lippek F Wollert-Wulf B. Increased myocardial expression of osteopontin in patients with advanced heart failure. Eur J Herat Fail. (2002) 4:139–46. 10.1016/S1388-9842(01)00237-9 11959041

[135] Singh K Sirokman G Communal C Robinson KG Conrad CH Brooks WW. Myocardial osteopontin expression coincides with the development of heart failure. Hypertension. (1999) 33:663–70. 10.1161/01.HYP.33.2.663 10024324

[136] Rosenberg M Zugck C Nelles M Juenger C Frank D Remppis A. Osteopontin, a new prognostic biomarker in patients with chronic heart failure. Circ Heart Fail. (2008) 1:43–9. 10.1161/CIRCHEARTFAILURE.107.746172 19808269

[137] Psarras S Mavroidis M Sanoudou D Davos CH Xanthou G Varela AE. Regulation of adverse remodelling by osteopontin in a genetic heart failure model. Eur Heart J. (2012) 33:1954–63. 10.1093/eurheartj/ehr119 21525025

[138] Schipper M Scheenstra MR van Kuik Y van Wichen DF van der Weide P Dullens FJ. Osteopontin: a potential biomarker for heart failure and reverse remodeling after left ventricular assist device support. J Heart Lung Transpl. (2011) 30:805–10. 10.1016/j.healun.2011.03.015 21531579

[139] Behnes M Brueckmann M Lang S Espeter F Weiss C Neumaier M. Diagnostic and prognostic value of osteopontin in patients with acute congestive heart failure. Eur J Heart Fail. (2014) 15:1390–400. 10.1093/eurjhf/hft112 23851388

[140] Tromp J Khan M Klip UT Meyer S de Boer R Jaarsma T. Biomarker profiles in heart failure patients with preserved and reduced ejection fraction. J Am Heart Assoc. (2017) 6:e003989. 10.1161/JAHA.116.003989 28360225 PMC5532986

[141] Becker P Waltenberger J Yachechko R Mirzapoiazova T Sham J Lee CG. Neuropilin-1 regulates vascular endothelial growth factor–mediated endothelial permeability. Circ Res. (2005) 96:1257–65. 10.1161/01.RES.0000171756.13554.49 15920019

[142] Wang Y Cao Y Yamada S Thirunavukkarasu M Nin V Joshi M. Cardiomyopathy and worsened ischemic heart failure in SM22-α cre-mediated neuropilin-1 null mice dysregulation of PGC1α and mitochondrial homeostasis. Arterioscler Thromb Vasc Biol. (2015) 35:1401–12. 10.1161/ATVBAHA.115.305566 25882068 PMC4441604

[143] Wei CM Lerman A Rodeheffer RJ McGregor C Brandt RR Wright S. Endothelin in human congestive heart failure. Circulation. (1994) 89:1580–6. 10.1161/01.CIR.89.4.1580 8149524

[144] Kiowski W Sütsch G Hunziker P Müller P Kim J Oechslin E. Evidence for endothelin-1-mediated vasoconstriction in severe chronic heart failure. Lancet. (1995) 346:732–6. 10.1016/S0140-6736(95)91504-4 7658874

[145] Koller B Steringer-Mascherbauer R Ebner C. Pilot study of endothelin receptor blockade in heart failure with diastolic dysfunction and pulmonary hypertension (BADDHY-Trial). Heart Lung Circ. (2017) 26:433–41. 10.1016/j.hlc.2016.09.004 27816421

[146] Packer M McMurray J Krum H. Long-term effect of endothelin receptor antagonism with bosentan on the morbidity and mortality of patients with severe chronic heart failure. JACC Heart Fail. (2017) 5:317–26. 10.1016/j.jchf.2017.03.003 28449795

[147] Djohan AH Sia CH Lee PS Poh KK. Endothelial progenitor cells in heart failure: an authentic expectation for potential future use and a lack of universal definition. J Cardiovasc Transl Res. (2018) 11:393–402. 10.1007/s12265-018-9810-4 29777508

[148] Cristóvão G Milner J Sousa P Ventura M Cristóvão J Elvas L. Improvement in circulating endothelial progenitor cells pool after cardiac resynchronization therapy: increasing the list of benefits. Stem Cell Res Ther. (2020) 11:194. 10.1186/s13287-020-01713-8 32448383 PMC7245793

[149] Berezin AE Kremzer AA. Relationship between circulating endothelial progenitor cells and insulin resistance in non-diabetic patients with ischemic chronic heart failure. Diabetes Metab Syndr. (2014) 8:138–44. 10.1016/j.dsx.2014.07.001 25082501

[150] Koller L Hohensinner P Sulzgruber P Blum S Maurer G Wojta J. Prognostic relevance of circulating endothelial progenitor cells in patients with chronic heart failure. Thromb Haemost. (2016) 116:309–16. 10.1160/TH16-01-0051 27412580

[151] Lee HJ Kim W Kim WS Woo JS Kim YG Moon JY. Circulating endothelial progenitor cell levels predict cardiovascular events in end-stage renal disease patients on maintenance hemodialysis. Nephron. (2015) 130:151–8. 10.1159/000430471 26089157

[152] Premer C Kanelidis AJ Hare JM Schulman IH. Rethinking endothelial dysfunction as a crucial target in fighting heart failure. Mayo Clin Proc Innov Qual Outcomes. (2019) 3:1–13. 10.1016/j.mayocpiqo.2018.12.006 30899903 PMC6408687

[153] Levi A Leshem-Lev D Weissler-Snir A Hasin T Mats I Murninkas D. The effect of mineralocorticoid receptor antagonists on recruitment and function of endothelial progenitor cells in patients with congestive heart failure. Isr Med Assoc J. (2018) 20:233–8. 29629731

[154] Tanaka S Sanuki Y Ozumi K Harada T Tasaki H. Heart failure with preserved vs reduced ejection fraction following cardiac rehabilitation: impact of endothelial function. Heart Vessels. (2018) 33:886–92. 10.1007/s00380-018-1128-2 29392470

[155] Dhingra R Vasan RS. Statistical assessment and section on key novel heart failure biomarkers. Trends Cardiovasc Med. (2017) 27:123–3. 10.1016/j.tcm.2016.07.005 27576060 PMC5253084

[156] Sponder M Lichtenauer M Wernly B Paar V Hoppe U Emich M. Serum heart-type fatty acid-binding protein decreases and soluble isoform of suppression of tumorigenicity 2 increases significantly by long-term physical activity. J Investig Med. (2019) 67:833–40. 10.1136/jim-2018-000913 30593542

[157] Mirna M Lichtenauer M Wernly B Paar V Jung C Kretzschmar D. Novel cardiac biomarkers in patients with cardiovascular diseases undergoing intensive physical exercise. Panminerva Med. (2020) 62:135–42. 10.23736/S0031-0808.20.03838-0 32309918

[158] Berezin AE Kremzer AA Martovitskaya YV Berezina TA Samura TA. The utility of biomarker risk prediction score in patients with chronic heart failure. Clin Hypertens. (2016) 22:3. 10.1186/s40885-016-0041-1 26973794 PMC4787185

